# Modified Glass Ionomer Cement with “Remove on Demand” Properties: An In Vitro Study

**DOI:** 10.3390/dj5010009

**Published:** 2017-01-23

**Authors:** Shaza Bishti, Taskin Tuna, Garima Agrawal, Andrij Pich, Stefan Wolfart

**Affiliations:** 1 Department of Prosthodontics and Biomaterials, Center for Implantology, Medical Faculty, RWTH Aachen University Hospital, Pauwelstrasse 30, 52074 Aachen, Germany; ttuna@ukaachen.de (T.T.); swolfart@ukaachen.de (S.W.); 2 Department of Polymer and Process Engineering, Indian Institute of Technology Roorkee, Saharanpur Campus, Paper Mill Road, Saharanpur- 247001, Uttar Pradesh, India; garimafpt@iitr.ac.in; 3 Functional and Interactive Polymers, DWI Leibniz Institute for Interactive Materials e.V., RWTH Aachen University, Forckenbeckstr. 50, 52074 Aachen, Germany; pich@dwi.rwth-aachen.de

**Keywords:** retrievability, glass ionomer cement, implant restorations, compressive strength

## Abstract

Objectives: To investigate the influence of different temperatures on the compressive strength of glass ionomer cement (GIC) modified by the addition of silica-coated wax capsules; Material and Methods: Commercially-available GIC was modified by adding 10% silica-coated wax capsules. Test blocks were fabricated from pure cement (control) and modified cement (test), and stored in distilled water (37 °C/23 h). The compressive strength was determined using a universal testing machine under different temperatures (37 °C, 50 °C, and 60 °C). The maximum load to failure was recorded for each group. Fractured surfaces of selected test blocks were observed by scanning electron microscopy (SEM); Results: For the control group, the average compressive strength was 96.8 ± 11.8, 94.3 ± 5.7 and 72.5 ± 5.7 MPa for the temperatures 37 °C, 50 °C and 60 °C respectively. The test group reported compressive strength of 64.8 ± 5.4, 47.1 ± 5.4 and 33.4 ± 3.6 MPa at 37 °C, 50 °C and 60 °C, respectively. This represented a decrease of 28% in compressive strength with the increase in temperature from 37 °C to 50 °C and 45% from the 37 °C to the 60 °C group; Conclusion: GIC modified with 10% silica-coated wax capsules and temperature application show a distinct effect on the compressive strength of GIC. Considerable compressive strength reduction was detected if the temperature was above the melting temperature of the wax core.

## 1. Introduction

One of the important decisions when placing implant-supported restorations is the choice of type of retention. Implant restorations may be either screw- or cement-retained. Several clinical and technical factors determine the choice of the suitable retention system [1]. These factors include: esthetic demands, ease of fabrication and cost, accessibility, retrievability, passivity of the framework and complications [2,3,4,5]. The outcome of both restorations has been well documented in the literature and reported no statistically significant difference between both. Nevertheless, each type of retention has its advantages and limitations [6,7,8].

Retrievability, which is sometimes required for peri-implant tissue assessment and maintenance of the prosthetic components, can be difficult and is therefore considered one of the main disadvantages of cement-retained restorations [6,9,10]. Although the survival rates of dental implants are increasing dramatically, the need for future removal of implant restorations should not be overlooked [11,12,13,14,15].

The selection of the cement for use in an implant-retained restoration should be based on the need or desire for retrievability, the anticipated amount of retention needed, the ease of cement removal and cost [16,17,18,19]. A variety of cements are currently available for restorative procedures. Most are primarily designed for teeth, and may be classified according to physical properties, material content and the purpose for which they were designed, for example, temporary or permanent cement. Some cements such as glass ionomer cement have unique properties. These include adhesion to dental tissue, biocompatibility and antibacterial activity [20]. Moreover, mechanical and bioactive properties have been successfully improved through the incorporation of nanofillers such as titanium dioxide (TiO_2_), synthesized hydroxyapatite and fluoroapatite nanoparticles [21,22,23].

Controversy exists as to whether a provisional or a permanent luting agent should be used. Some studies recommended the use of temporary cements for the retention of implant restorations [24,25]. This facilitates retrievability when required without damaging the restoration. However, this advantage is accompanied by poor physical properties, in terms of low tensile strength and high solubility. The risk of retention loss can be reduced by the use of permanent cements. Nevertheless, due to their permanent properties, removal of the superstructure when needed is almost impossible to achieve without causing harm to the implant restoration components and the peri-implant tissues [16,26].

Therefore, a method of semi-permanent retention that can provide adequate retention and allows retrievability whenever required would be desirable. Several methods for achieving such method have been reported in the literature. Some authors recommended weakening of the cement by mixing it with petroleum jelly [27]. Others suggested placement of a retrieval screw threaded into the implant restoration in a location in which a displacing force can be applied to break the cement seal [28,29,30]. Another suggested attempt to retrieve cemented implant restorations is through certain design features (e.g., lingual groove, connectors). However, it is unclear which kind of the above-mentioned methods is useful to achieve adequate retention and maintain retrievability.

One option that still has not been considered so far is the possibility of controlled destruction of dental cements, giving cements “remove on demand” properties. Cements with such properties are not available in the market and not present in the scientific literature.

The general aim of this project is to modify properties of commercial cements and to develop dental cements with so-called “remove on demand” (ROD) properties that can be used for implant-supported restorations. The idea is to add activatable micro-additives that can reduce the mechanical properties of the cement by application of an external stimulus (temperature, ultrasound, magnetic field or light energy). This may allow fast, painless and non-destructive removal of the restoration from the implant. Therefore, designing biocompatible non-toxic sensitive nanoscopic building blocks with tunable properties and integrating them into a dental cement matrix is the challenge.

**Figure 1:**
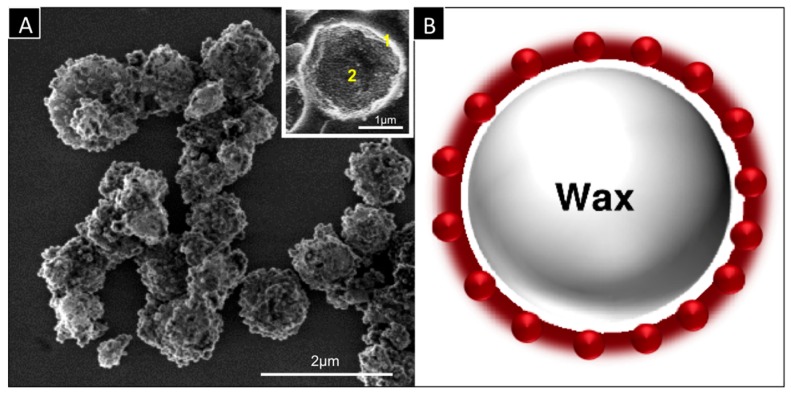
(**A**) SEM image of a smart capsule with a diameter of 1–3 μm, showing (1) silica capsule wall with a thickness of 0.15–0.3 μm and (2) docosane wax core with a melting range of 45–47 °C; (**B**) Schematic representation of cross-sectional area of a smart capsule.

The key technology of the research work is a smart capsule (SC) consisting of a thin wall that allows fast heat transfer and a core filled with a phase change material (PCM) able to undergo a phase transition (solid-liquid) upon heating (Figure 1). The coupling of both elements allows rapid transformation of solid core into liquid core capsules under elevated temperatures and thus considerable reduction of their mechanical properties. If such capsules are integrated as fillers into a solid matrix (dental cement), temperature increase will induce rapid weakening of the cement through formation of voids or cracks thus allowing its destruction. Accordingly, this project is divided into 4 main parts: (a) fabrication of the capsules; (b) optimizing the cement properties; (c) preclinical tests and (d) clinical tests.

The first part of the research work, which includes fabrication of smart capsules, has already been performed in a previous study [31]. In this work, smart capsules were prepared based on Pickering emulsion and sol-gel process. It was concluded that these capsules could be further used for developing remove on demand dental cements. According to the results of this study, it is expected that local heating will destabilize the modified cement and the restoration can be removed without any damage. After achieving promising results with the first step of the project (capsule fabrication), the specific aim of this in vitro study was to investigate the influence of different temperatures on the compressive strength of dental cement modified by the addition of smart capsules.

## 2. Materials and Methods

Glass ionomer cement “GIC” (Vivaglass CEM PL, Ivoclar Vivadent AG, Schaan, Liechtenstein) was used in this in vitro study. The study design was to compare the compressive strength properties of a control group consisting of test blocks made of pure cement (with no additives), with a test group made from modified cement mixed with additives (smart capsules).

### 2.1. Preparation of Smart Capsules and Incorporation within the Cement

For the fabrication of capsules, tetraethoxysilane (TEOS) (99%, VWR International GmbH, Darmstadt, Germany), ethanol (99.9%, VWR), ammonia (NH_3_·H_2_O, 25%), hexadecyltrimethoxysilane (HDTMS) (90%, ABCR), docosane (99%, Sigma-Aldrich Chemie GmbH, Munich, Germany) and poly(diethoxysiloxane) (TEOS-40) (Si = 20.5%–21.5%, ABCR) were used as received. As a PCM, docosane wax (Sigma-Aldrich Chemie GmbH, Munich, Germany) with a melting range of 42–45 °C was used.

Smart capsules were prepared according to the literature procedure [32]. Briefly, molten wax (0.4 g) and TEOS-40 silica precursor (0.4 g) were added to 10 g aqueous silica dispersion consisting of 0.12 g silica nanoparticles followed by ultrasonic emulsification for 15 min (Branson Sonifier 450 cell disrupter, 3 mm microtip, 247 W output). The obtained emulsion was gently stirred for three days at room temperature. The capsules were purified by three repeated cycles of centrifugation and washed with water.

In order to identify the best amount of smart capsules to be incorporated within the cement, several pretests with the aid of dummy particles were performed. The results of these tests indicated the use of a percentage of 10% smart capsules. For incorporation, the glass ionomer cement powder as well as the smart capsules were accurately weighed with the aid of a digital scale (Sartorius ME215S, Sartorius Weighing Technology GmbH, Goettingen, Germany). The cement powder was mixed in small glass containers using a shaker (Heidolph Multi Reax, Heidolph Instruments GmbH & Co. KG, Schwabach, Germany) with 2000 U/min for 60 min to ensure complete incorporation of the particles within the cement.

### 2.2. Mixing

**Figure 2:**
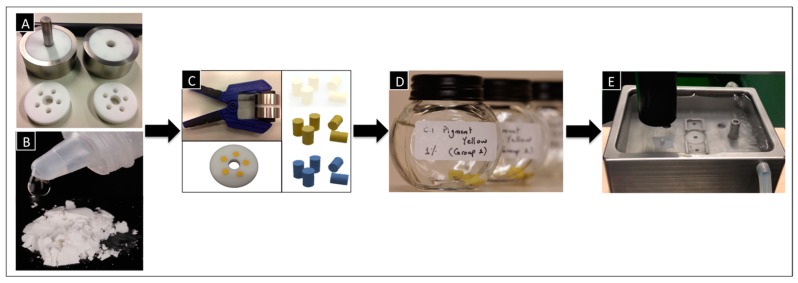
Workflow for the fabrication of the test blocks: (**A**) The Teflon discs engaged in a metal housing with a Teflon disc in between; (**B**) Cement mixing; (**C**) Molding press holding the discs containing the test blocks, then 1 h after setting; (**D**) Test blocks stored in distilled water at 37 °C; (**E**) Compressive strength tests using the universal testing machine (UTM).

Both cement groups (control and test) were hand mixed according to the manufacturer’s instructions. The range of powder/liquid mixing ratio was previously identified (Figure 2B), then mixed by spatulation. The powder and liquid were mixed to a homogenous, creamy consistency using a plastic spatula.

### 2.3. Fabrication of Test Blocks

To fabricate the test blocks, special molds had to be first prepared. The mold consisted of three pieces, including two Teflon discs, each engaged in a metal housing, and a third Teflon disc in between. The middle Teflon disc had five holes, which facilitated the fabrication of five test blocks simultaneously. Teflon was used to ease the removal of the test blocks from the discs later. The holes had internal dimensions of 6 mm in height and 4 mm in diameter (Figure 2A).

Within 60 s after the end of mixing, the cement was applied using a special plastic syringe into the mold holes of the Teflon disc. After the excess cement was removed, the Teflon disc was pressed between the two corresponding metal discs with the aid of a molding press (50 N) and put directly in the oven (37 °C) for 1 h (Figure 2C).

After 1 h, any surplus cement was removed by polishing both sides of the Teflon mold with 800-grit silicone carbide paper. The finally obtained test blocks had a height of 6 mm and diameter of 4 mm The cement test blocks were carefully removed from the molds and stored in distilled water at 37 °C for another 23 h (Figure 2D). Figure 2 shows the whole workflow for the fabrication of the test blocks.

**Figure 3:**
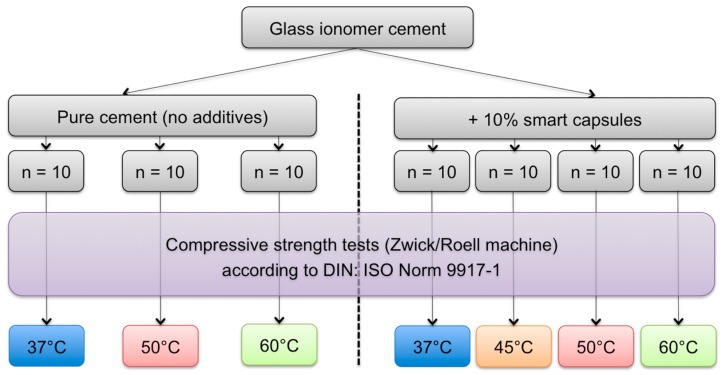
A graph illustrating the different control and test groups subjected to compressive strength tests. A total of 30 test blocks were used for each group with temperatures 37 °C, 50 °C and 60 °C. Ten extra blocks were compressed under 45 °C for the test group to investigate if there was a decrease in compressive strength in temperatures lower than 50 °C.

A total number of 70 test blocks were fabricated and divided into different groups as shown in Figure 3.

### 2.4. Compressive Test

Twenty-four hours after the end of mixing, each test block was removed from the storage water and its diameter and height was measured using a micrometer. The compressive strength of the blocks was determined according to DIN: ISO Norm 9917-1 using a mechanical testing machine, Zwick/Roell machine, software version: TestXpert II (Zwick GmbH & Co. KG, Ulm, Germany) at a test speed of 0.75 mm/min. A water bath was used to achieve the desired temperatures (37 °C, 45 °C, 50 °C and 60 °C), and filter paper was placed under each test block (Figure 2E). To assure that the test blocks reached the temperature needed, the blocks were left in the water bath for 5 min before the measurements started. The maximum load to failure was recorded and every series of measurements consisted of 10 identical test blocks to reduce random errors.

The compressive strength, C (MPa), of each individual cylindrical block was calculated using the following equation:

$$C=\frac{4\;p}{\pi \;d^{2}}$$

where *p* is the largest applied force in Newton and *d* is the diameter of test blocks, in millimeters.

### 2.5. SEM Observation

Following the compressive strength tests, fractured surfaces of selected test blocks were observed by scanning electron microscopy (FE-SEM, Hitachi S-4800, Tokyo, Japan) at an operating voltage of 5 kV at low (5000×) and high (20,000×) magnifications. The fractured surfaces were observed to investigate the distribution and the surface characteristics of the capsules within the cement matrix.

### 2.6. Statistical Analysis

Statistical analyses were performed using SPSS Statistics 19 (version19.0.0, SPSS Inc., Chicago, IL, USA). Beyond standard descriptive statistics, inductive analyses were performed using non-parametric methods. For all between-group analysis (control and test groups under different temperatures), the Kruskal-Wallis-Test was used. This was followed by multiple comparisons using the Mann-Whitney-U-Test adjusted with the Bonferroni Holm procedure. The significance level was set at α = 0.05.

## 3. Results

The results for the compressive strength tests under different temperatures for the pure cement group and the smart capsule group are shown in details in Table 1. It is noteworthy to mention that before undergoing the compressive strength tests, visual observation of the test blocks after removal from the molds indicated the presence of cracks, which were confirmed later by the SEM images.

For the control group (with no additives), a decrease of 4% in compressive strength with the increase in temperature from 37 °C to 50 °C was reported. This was considered statistically insignificant (*p* = 0.415). Increasing the temperature up to 60 °C showed a statistically significant difference (*p* ≤ 0.001) and revealed a compressive strength decrease of 26% from the 37 °C group.

**Figure 4:**
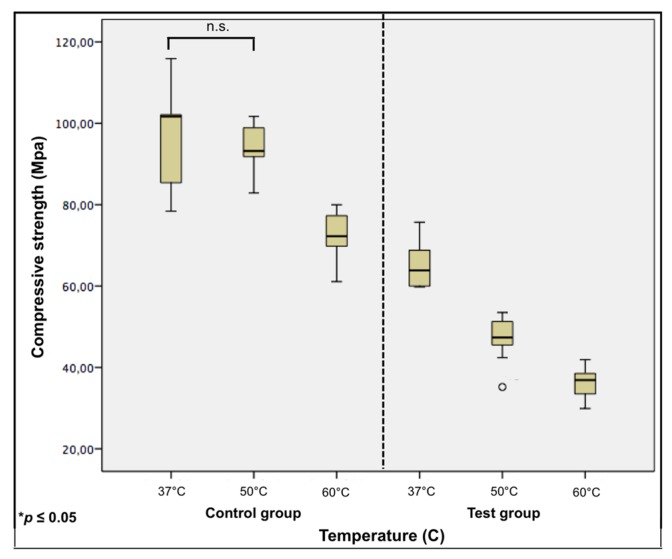
The compressive strength of groups under different temperatures, represented in box plots. All groups reported a statistically significant difference between one another, except the control 37 °C with control 50 °C group. Non-significant differences are connected by the horizontal lines (n.s.).

For the test group, changes in the compressive strength represented a decrease of 28% with the increase in temperature from 37 °C to 50 °C. Increasing the temperature up to 60 °C revealed a compressive strength decrease of 45% from the 37 °C group. All showed a statistically significant difference to one another (*p* ≤ 0.001). Here, an extra group (*n* = 10 test blocks) was tested under a temperature of 45 °C to investigate whether a significant decrease in the compressive strength already occurred in temperatures lower than 50 °C. This test reported an average compressive strength of 62 MPa, which represented a decrease of 4% from the 37 °C group and was considered statistically insignificant (*p* = 0.912). Boxplots with the compressive strength of both groups under different temperatures with computed statistical comparisons are shown in Figure 4.

**Figure 5:**
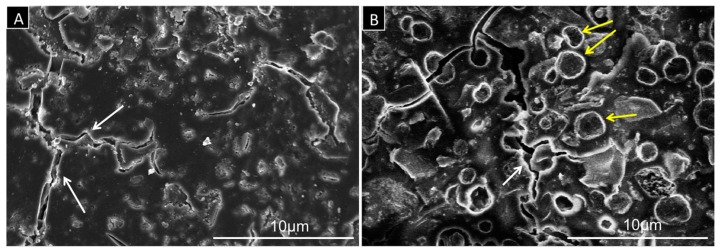
SEM images at 5000× magnification of (**A**) pure cement (control group); and (**B**) modified cement (10% smart capsules). Microcracks with a width of 0.1–2 µm are clearly visible in both groups (white arrows). The modified cement shows relatively well-distributed spherical structures representing the smart capsules (yellow arrows).

Figure 5 shows examples of SEM images for fractured surfaces in control and test blocks equilibrated in water at a physiological temperature of 37 °C. For both samples, the images indicate presence of cracks with a width range of 0.1–2 µm. The SEM image of the modified cement (test group) shows relatively well-distributed spherical structures embedded in the cement matrix, representing the smart capsules. The capsules show a well-defined outline with a wall thickness of 0.15–0.3 µm.

**Figure 6:**
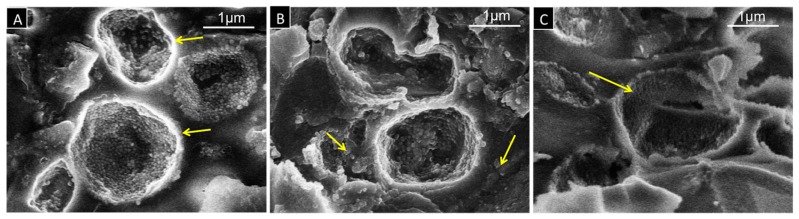
SEM images at 20,000× magnification of modified cement, representing the structural changes after treatment at: (**A**) 37 °C showing spherically shaped intact capsules filled with wax (yellow arrows); (**B**) 50 °C with capsules filled with a less amount of wax and grain-like wax structures outside the capsules (yellow arrows); (**C**) 60 °C with broken empty capsules (yellow arrow).

Figure 6 shows SEM images for fractured surfaces in test blocks treated at different temperatures to illustrate the structural changes of the capsule morphology under heat treatment. In Figure 6A, three capsules on the fractured surface of the test block treated at 37 °C are displayed. At high magnification, the images show that capsules are intact and filled with wax. Figure 6B represents the morphology of the capsules after treatment at 50 °C. In this sample, capsules are filled with a less amount of wax and some grain-like wax structures outside the capsules are visible. Figure 6C shows broken, hollow and irregularly shaped empty capsules in the sample treated at 60 °C.

## 4. Discussion

This in vitro study showed that the mean compressive strength of the cement decreased with the addition of specific additives formulated in form of wax core silica shell capsules. This indicates that variation of the internal structure of dental cement has a causative effect on its mechanical properties. In addition, temperature is used as a physical trigger to modulate the compressive strength of modified cements.

In this in vitro study, glass ionomer cement was used since it has been well documented in the literature, and is widely used in the dental practice as a semi-permanent dental cement [32].

The results of the current in vitro study showed that a temperature of 50 °C was enough to destroy the capsules leading to a significant drop in the compressive strength of the cement, and the pressure needed to break the test blocks was much lower than that for the 37 °C group. In order to make sure that the cement strength was not affected at lower temperatures, compressive strength tests were done at 45 °C, which is within the melting range of the wax used for capsule fabrication (Docosane, melting range 42–45 °C). Here, a compressive strength decrease of 3.68% from the 37 °C group occurred and was considered statistically insignificant. This reveals that the melting range of wax here is slightly wider than for pure wax due to its incorporation in small volume within a silica shell. A similar effect was observed by Agrawal et al. [33]. This can be explained by the modification of wax due to the incorporation of silica to the structure of capsules.

Identifying the most suitable amount of capsules incorporated within the cement was first achieved. For this, pre-studies investigating the compressive strength of the cement mixed with dummy particles were performed. Different amounts (1%, 5%, 10%, 20%) were used and compressive strength tests of composite cements were undergone. With additives, the capsules should be homogenously distributed within the cement and the compressive strength must remain within the requirements for industrial application in order to be clinically used for its purposes. This was investigated by the SEM images, which indicated very good compatibility of the capsule surface with the glass-ionomer cement, which ensures good mechanical properties of composites before temperature treatment. Samples with 10% dummy particles reported the highest compressive strength among all percentages examined in this stage. Here, the lowest value was set at 50 MPa according to the norm EN ISO 9917-1. In addition, this amount of additives had no negative influence on the mixing and handling of the cement. Moreover, the working and setting time were similar to the pure GIC. According to these pretest results, the current study was performed using 10% smart capsules.

Because of the obvious loss of compressive strength with 10% and due to the negative results achieved from pretests performed with 20% dummy particles, higher percentages of capsules were not used. Moreover, the use of 20% dummy particles had negatively influenced the practical properties of GIC. Mixing and handling difficulties due to insufficient combination of the cement powder with the liquid, as well as decreased working time due to increased mixing time were the man problems encountered.

Recent studies reported variations of composite materials properties depending on powder/liquid ratio, mixing technique and variances in operator [34,35,36,37]. In an attempt to eliminate as much as possible the variable factors that may affect the results of this study, care was taken to identify the ideal powder/liquid ratio as well as the best mixing technique for the cement. Here, both powder and liquid were measured carefully with the aid of a digital scale, and mixed in a ratio of 3:1 according to the manufacturer’s instructions. In terms of cement mixing technique, the modified cement used in the current study showed incomplete incorporation of the powder within the liquid when mixed by rotation, therefore, hand mixing was chosen for mixing both groups of cement used in this study. In addition, all investigations were performed by the same operator.

Regarding the behaviour of pure GIC to different temperatures, increasing the temperature to 50 °C revealed no significant drop in the compressive strength of the cement. However, 60 °C was enough to show a significant decrease in the cement’s compressive strength. This may be related to the structure of the cement itself. The structure of the set GIC has been described as a matrix consisting of calcium and aluminium polyacrylates with glass particles embedded in it surrounded by a siliceous hydrogel [38,39]. The drop in compressive strength of the cement was proposed to be due to increased cross-linking of the silica gel producing some brittleness of the set cement [40]. However, the results of our study did not show similarity to other studies, which reported no significant difference in compressive strength after heat treatment [41,42]. An increase in the mechanical properties of the cement was mainly found to occur in resin-modified GICs and not in conventional cements [43]. For the cement modified with smart capsules, the results of this study indicate that 50 degrees is enough to destruct the capsules thus further reducing the compressive strength of the cement. The strength of the modified cement was almost half that of the pure cement under a temperature of 50 °C. The structural changes within the cement prove that at high temperatures capsules get weaker and become destroyed under mechanical load, which leads to the weakening of compressive strength of the composite cement.

However, from a clinical point of view, several questions still remain open and have to be answered. These include: how would food intake with different temperatures influence the cement? What is the maximum temperature a subject can withstand in the mouth e.g., when drinking hot beverages? How long does it take for these beverages to cool down intraorally? How long do these hot beverages remain in contact with intraoral tissues? In this context, a limited number of relevant studies were found in the literature. One study investigating the coffee temperature preferred by consumers and the effect of these temperatures on intraoral tissues reported that the preferred temperatures for drinking ranged between 37.6 °C and 88 °C with a mean of 59.8 °C. These temperatures were not only above reported pain thresholds but also above possible damage thresholds in the mouth [44]. So, why don’t people burn their mouths or suffer from pain when drinking hot beverages at their preferred temperatures? Although there was a drop in temperature of served beverages entering the oral cavity, this temperature drop was insufficient to prevent pain and tissue damage and still exceeded the human thermal pain threshold. This indicated that the hot beverages never contacted the intraoral epithelial surfaces long enough to induce pain or tissue damage [44]. This may also indicate that hot beverages would have no effect on restorations cemented with the modified dental cement represented in this in vitro study. In addition, retrievability of these restorations should be performed with a special device that can be applied directly on the restoration in order to transfer heat locally with no effect or damage to the surrounding tissues and with limited heat transmission through the restoration and implant to the periimplant bone. Here, it is noteworthy to mention that this project idea is planned only for cement-retained implant restorations and not for conventional ones (tooth-supported).

In order to reinforce the results of this study and to investigate whether this technique is reliable and can be clinically used, further preclinical studies in terms of tensile strength tests in combination with chewing simulation and thermocycling on restorations simulating the intraoral situation are currently under investigation.

## 5. Conclusions

This in vitro study has shown that variable amounts of capsules can be incorporated into GIC forming a composite material. The capsules were well distributed and showed good compatibility with the cement matrix. GIC samples modified with 10% of capsules showed to have the most promising results. With this amount of additives, considerable reduction of the compressive strength was detected if the temperature was above the melting temperature of the wax core. Considering this, we conclude that solid-liquid transition of the capsule core triggered by temperature is ultimately influencing the compressive strength of modified GIC cements. We believe that this approach can be used to fabricate new dental cements able to tune their mechanical properties under temperature variation.

## Figures and Tables

**Table 1:** Compressive strength (MPa) of both control and test groups at different temperatures (37 °C, 50 °C and 60 °C).

Group	Temperature	Mean (MPa) ± Standard Deviation	Max.	Min.	Median	25th Percentile	75th Percentile
Control	37 °C	96.8 ± 11.8	115.9	78.4	101.7	84.7	103.3
	50 °C	94.3 ± 5.7	101.7	82.9	93.2	91.5	99.5
	60 °C	72.5 ± 5.6	80	61.1	72.2	69.6	77.8
Test	37 °C	64.8 ± 5.4	75.7	59.8	63.8	59.9	69.2
	45 °C	62.5 ± 2.8	70.6	39.7	66.9	59.1	67.2
	50 °C	47.1 ± 5.4	53.5	35.2	47.3	44.7	51.6
	60 °C	33.4 ± 3.6	41.9	29.9	36.9	33.0	38.7
